# Dose-Dependent Hepatotoxicity of Zinc Oxide Nanoparticles in Rats: Oxidative Stress, Apoptosis, and Dysregulation of Hepatic miR-122, miR-34a, and miR-21

**DOI:** 10.3390/ijms27188073

**Published:** 2026-09-10

**Authors:** Rasha Muzahem Hatem

**Affiliations:** Department of Biology, College of Science, University of Al-Qadisiyah, Al-Diwaniyah 58001, Iraq; rasha.albukhlate@qu.edu.iq

**Keywords:** zinc oxide nanoparticles, hepatotoxicity, oxidative stress, apoptosis, inflammatory signaling, microRNAs

## Abstract

Zinc oxide nanoparticles (ZnO NPs) are widely used in biomedical, agricultural, and industrial applications, raising concerns about their potential hepatotoxicity. This study investigated the effects of 28-day oral ZnO NP exposure on hepatic function, oxidative stress, apoptosis, inflammatory signaling, and microRNA expression in rats. Sixty male Wistar albino rats were allocated to three groups (*n* = 20 each),control, low dose (30 mg/kg), and high dose (100 mg/kg), and treated by oral gavage. The administered material had a nominal particle size of 50 nm and a supplier-stated purity of 99.9%. Serum alanine aminotransferase (ALT) and aspartate aminotransferase (AST), hepatic malondialdehyde (MDA) and reduced glutathione (GSH), histopathology, immunohistochemistry, mRNA expression, and hepatic microRNAs were assessed. ZnO NPs induced dose-related liver injury: ALT increased from 31.26 ± 2.5 to 75.48 ± 12.3 IU/L, while AST increased from 32.47 ± 4.1 to 85.57 ± 18.9 IU/L. Hepatic MDA increased, whereas GSH increased at the low dose but was depleted at the high dose. Histopathology demonstrated sinusoidal congestion, hepatocellular necrosis, and inflammatory infiltration. Immunohistochemistry indicated reduced B-cell lymphoma 2 (Bcl-2) and increased caspase-3 and tumor necrosis factor-alpha (TNF-α) immunoreactivity. Interleukin-6 (IL-6) and heme oxygenase-1 (HO-1) were upregulated, whereas superoxide dismutase 2 (SOD2) was downregulated. At the high dose, miR-122-5p, miR-34a-5p, and miR-21-5p increased by 8.74-, 6.53-, and 4.38-fold, respectively (all *p* < 0.05). Their expression levels were also strongly and positively correlated with biochemical, oxidative, and histopathological indicators of liver injury. These findings indicate that ZnO NP exposure is associated with oxidative, inflammatory, apoptotic, and microRNA responses; the altered hepatic microRNAs may represent candidate tissue indicators of ZnO NP-induced liver injury.

## 1. Introduction

Nanotechnology has emerged as a rapidly advancing field concerned with the design, synthesis, and application of materials at the nanoscale, typically defined as structures ranging between 1 and 100 nm [1,2]. Owing to their small size and exceptionally large surface-area-to-volume ratio, nanoparticles exhibit a remarkable capacity to interact with biomolecules at and within cell surfaces, which underlies their widespread use across the life sciences, medicine, biotechnology, and veterinary medicine [3,4].

Because of their dimensions, nanoparticles can cross biological barriers, traverse the intestinal epithelium, and reach the bloodstream, from which they distribute to multiple organs, including the lungs, brain, heart, spleen, kidneys, and liver [5,6]. Human and animal exposure occurs through several routes, principally inhalation, dermal contact, and the gastrointestinal tract [7,8,9]. The biological behavior of nanomaterials is governed by their large surface area, high reactivity, and chemical stability [10,11], and is further modulated by physicochemical factors such as chemical composition, shape, surface charge, and particle size [12,13].

Zinc is an essential trace element and a cofactor for numerous enzymes, including copper–zinc superoxide dismutase (Cu/Zn-SOD), a key antioxidant enzyme. It participates in a broad range of physiological processes, including the antioxidant defense system, immune function, protein and DNA synthesis, growth and development, thyroid metabolism, nerve conduction, and wound healing [14,15]. Among metal oxide nanomaterials, zinc oxide nanoparticles (ZnO NPs) are among the most extensively produced and studied, alongside gold, silver, and iron oxides [10,16], owing to their favorable physicochemical properties, functional versatility, and ease of manufacture [10,17]. Consequently, they are widely incorporated into industrial and commercial products, medicine, antibacterial agents, cancer therapeutics, cosmetics, agriculture, and food [18].

However, the increasing production and environmental release of ZnO NPs increase the likelihood of exposure in living organisms, while their toxicity has received comparatively little attention [19], prompting growing concern regarding their potential adverse effects [2,5]. Despite their ease of cellular penetration and therapeutic benefits, ZnO NPs have been shown to induce cytotoxicity in various organs, largely through the generation of oxidative stress [20]. The liver, as the principal organ responsible for metabolism, detoxification, and xenobiotic clearance, is particularly vulnerable, given its continuous exposure to nanoscale materials through dietary intake, pharmaceuticals, and environmental pollutants [21,22]. This makes the liver a critical target for evaluating ZnO NP-induced toxicity.

In parallel, microRNAs (miRNAs), small non-coding RNAs that regulate gene expression post-transcriptionally, have emerged as sensitive molecular indicators of hepatic injury, often reflecting cellular damage earlier and more specifically than conventional biochemical markers [23,24]. Among them, miR-122 is a liver-enriched miRNA and an established biomarker of hepatocellular injury; miR-34a is a p53-responsive, pro-apoptotic miRNA; and miR-21 is a stress- and inflammation-associated miRNA implicated in hepatic survival and inflammatory signaling [24,25]. The coordinated dysregulation of these three miRNAs may therefore capture the oxidative, apoptotic, and inflammatory dimensions of nanoparticle-induced liver injury within a single molecular framework.

Despite accumulating evidence of ZnO NP hepatotoxicity, few studies have integrated conventional biochemical, histopathological, and immunohistochemical endpoints with hepatic miRNA profiling within a single dose–response model. Therefore, the present study aimed to investigate the dose-dependent effects of subacute (28-day) oral exposure to ZnO NPs on liver function, oxidative stress, apoptosis, and inflammatory signaling in male rats, and to determine whether hepatic miR-122, miR-34a, and miR-21 expression parallels the severity of injury and could serve as sensitive biomarkers of ZnO NP-induced hepatotoxicity.

## 2. Results

Oral administration of ZnO NPs for 28 days induced dose-dependent alterations in hepatic function, oxidative status, gene expression, and microRNA profiles, as detailed below.

### 2.1. Liver Enzymes (ALT and AST)

Exposure to ZnO NPs caused a significant, dose-dependent elevation in serum liver enzymes (ALT and AST) compared with the control group (Table 1). Enzyme activities increased in both the low- and high-dose groups, with the highest values recorded in the high-dose group. These findings indicate progressive hepatic injury and impaired liver function following 28-day exposure to ZnO NPs.

### 2.2. Oxidative Stress Markers (MDA and GSH)

ZnO NPs induced marked alterations in hepatic oxidative status (Table 2). Malondialdehyde (MDA) levels were significantly increased in both treated groups, particularly in the high-dose group, indicating enhanced lipid peroxidation and oxidative damage. In contrast, reduced glutathione (GSH) showed a biphasic response, with significantly higher levels in the low-dose group and significantly lower levels in the high-dose group compared with controls.

### 2.3. Hepatic Gene Expression (IL-6, SOD2, HO-1)

**Figure 1:**
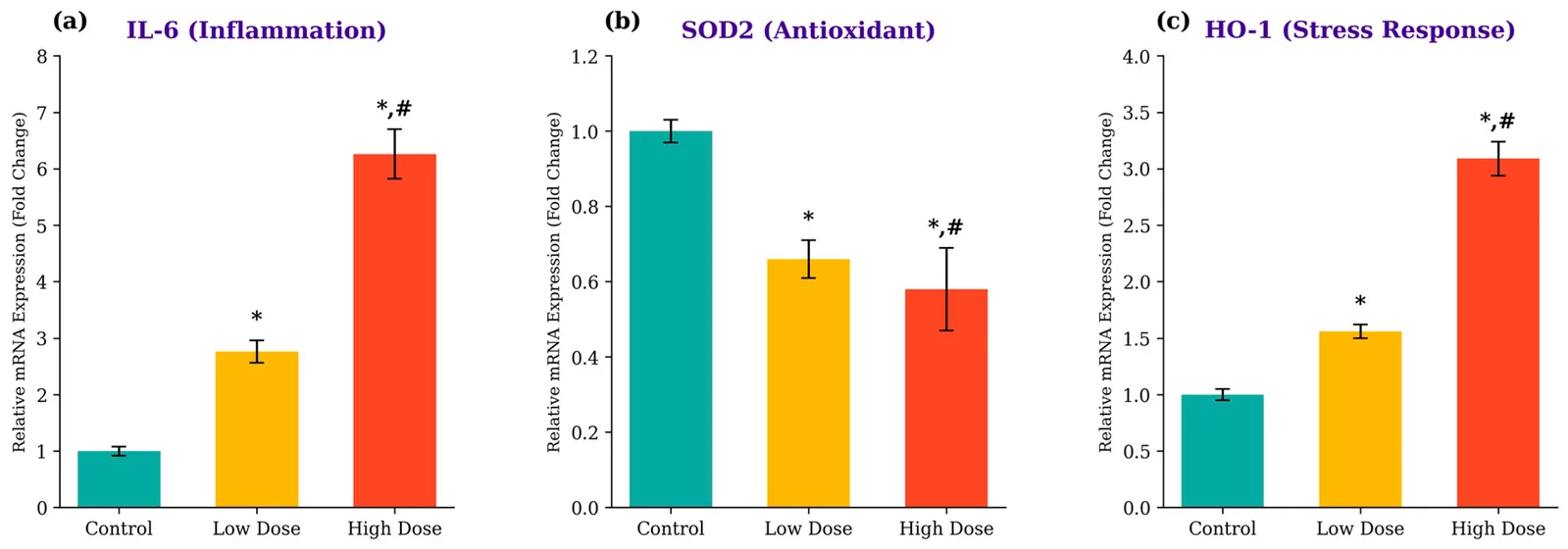
Hepatic gene expression of IL-6, SOD2, and HO-1 in rat liver following 28-day exposure to ZnO nanoparticles. (**a**) IL-6; (**b**) SOD2; (**c**) HO-1. Relative expression was calculated using the 2−ΔΔCt method, with the control group normalized to 1.00 for each target gene. IL-6 (inflammation) and HO-1 (stress response) were upregulated, whereas SOD2 (antioxidant) was downregulated. Values are expressed as mean fold change ± SEM (*n* = 20/group). * *p* < 0.05 vs. control; # *p* < 0.05 vs. low-dose group. One-way ANOVA followed by Tukey’s post hoc test.

Molecular analysis revealed significant changes in hepatic gene expression (Figure 1). For each target gene, the control group was used as the calibrator and normalized to 1.00. The pro-inflammatory cytokine IL-6 and the stress-response enzyme HO-1 were markedly upregulated in ZnO NP-treated rats, especially in the high-dose group, indicating activation of inflammatory and oxidative stress pathways. Conversely, SOD2 expression was significantly reduced in both treated groups relative to the control, indicating impaired mitochondrial antioxidant defense.

### 2.4. Histopathological Findings

**Figure 2:**
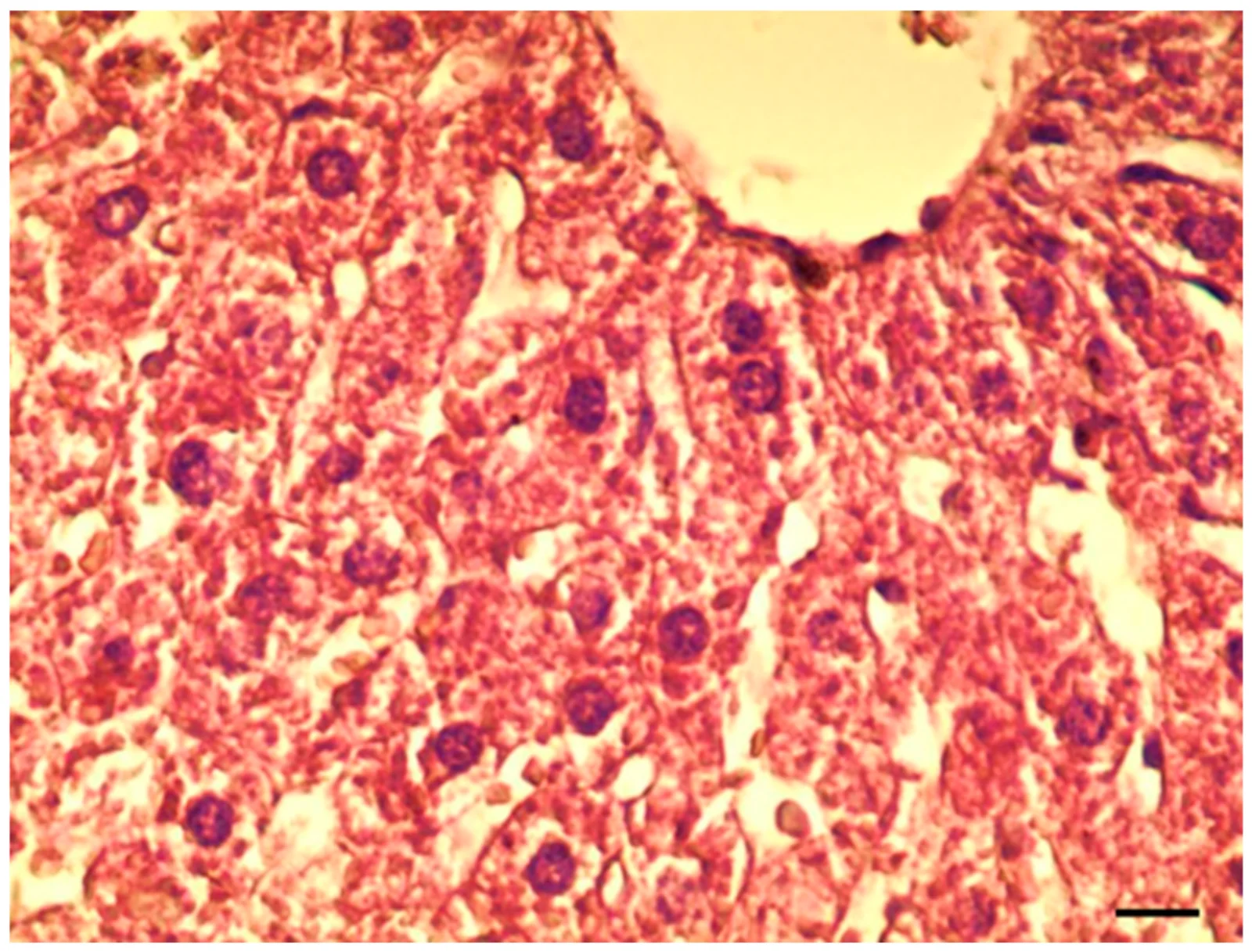
Control group showing normal hepatic architecture, with hepatocytes arranged in cords radiating from the central vein and separated by hepatic sinusoids. H&E staining, ×400. Scale bar = 50 µm.

**Figure 3:**
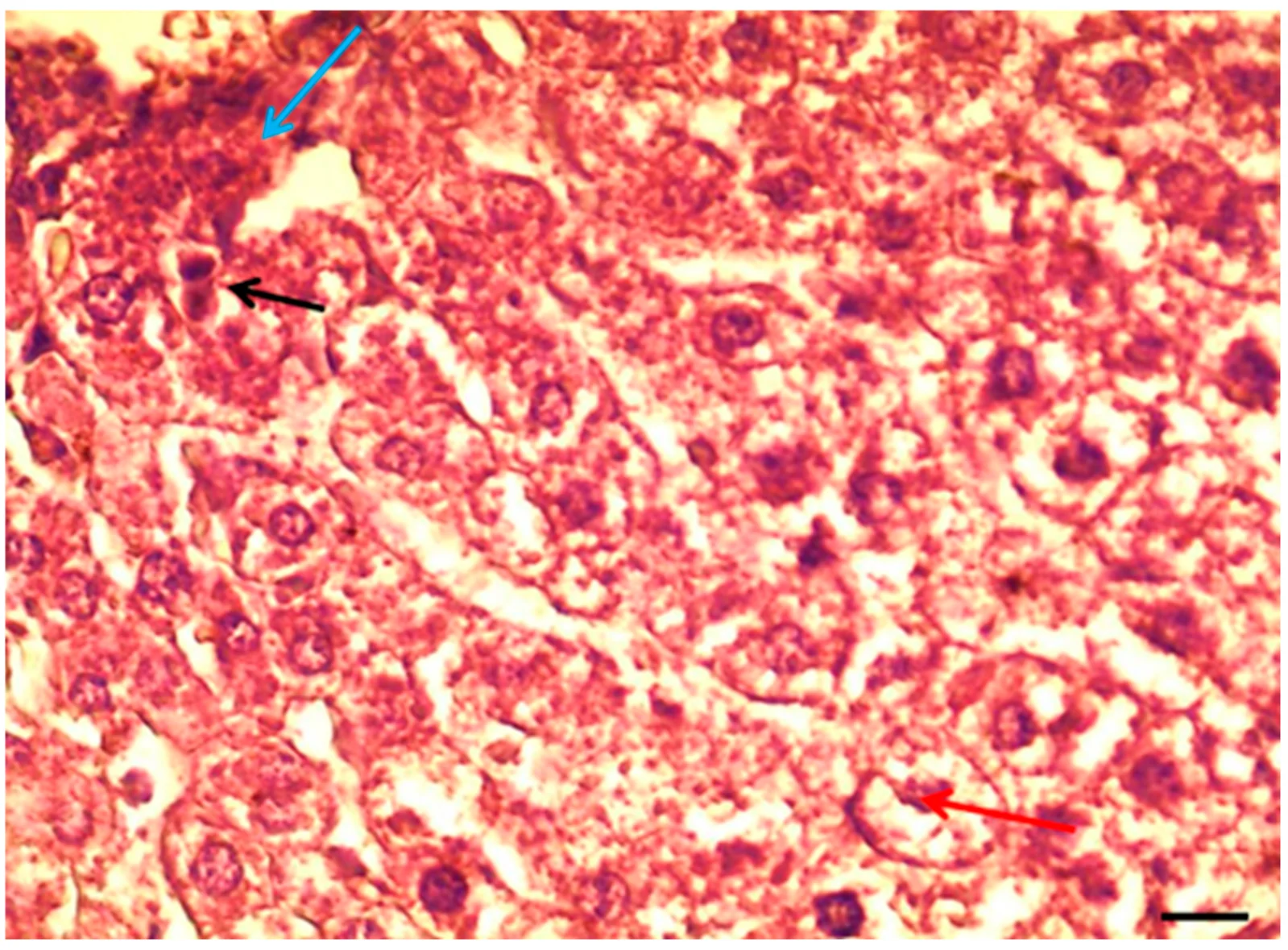
Low-dose group (30 mg/kg). Liver section showing mild sinusoidal congestion, focal hepatocyte necrosis, and mild lymphoid infiltration in the portal area, with largely preserved hepatic architecture. Black arrow, hepatocytes arranged in cords separated by hepatic sinusoids; blue arrow, mild vascular congestion; red arrow, focal hepatocyte necrosis. H&E staining, ×400. Scale bar = 50 µm.

**Figure 4:**
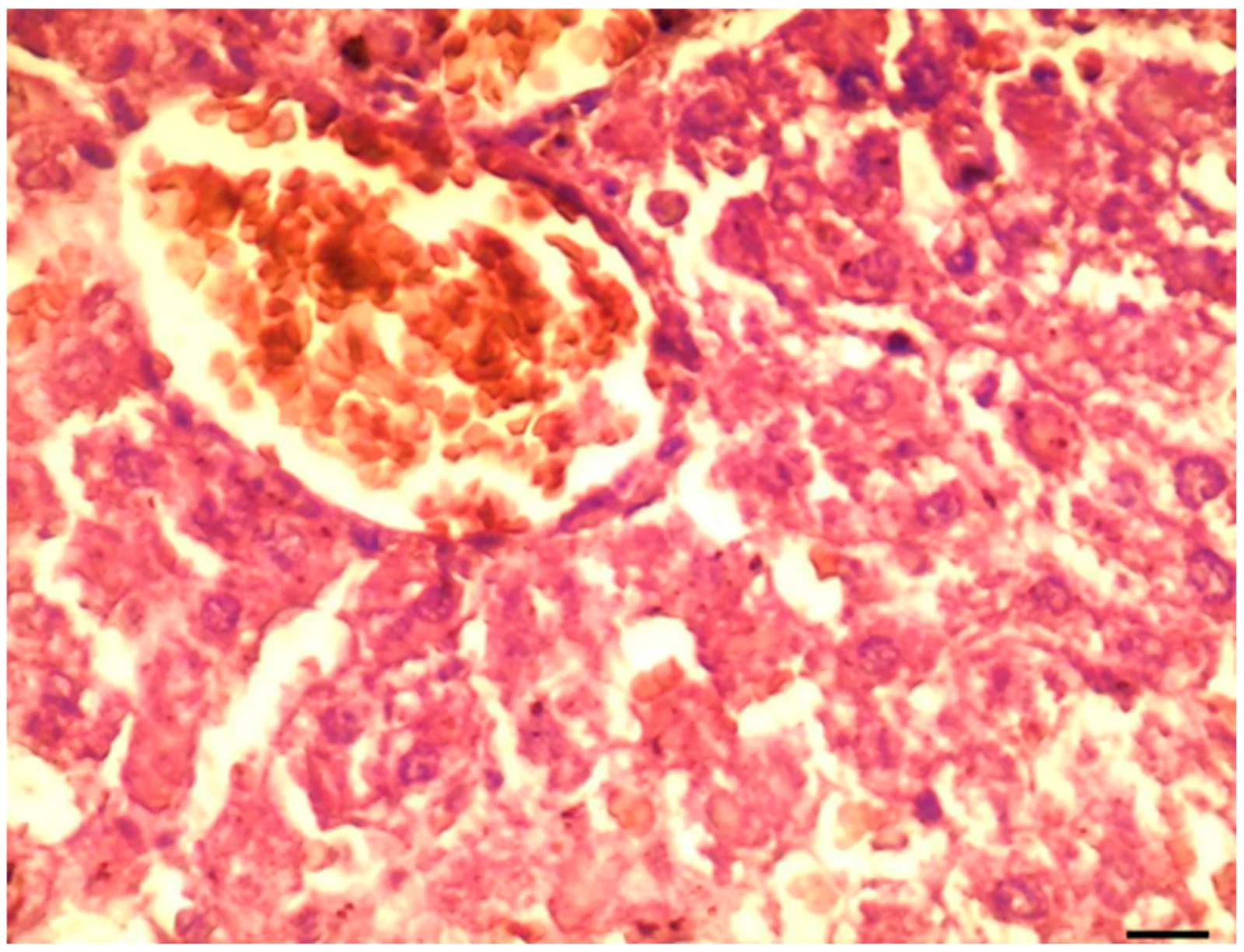
High-dose group (100 mg/kg). Liver section showing marked disruption of hepatic architecture, with loss of the normal cord arrangement, vascular congestion, extensive hepatocyte necrosis, and inflammatory cell infiltration. H&E staining, ×400. Scale bar = 50 µm.

Histological examination revealed dose-dependent structural damage in ZnO NP-treated rats. Control livers showed normal hepatic architecture, with hepatocytes arranged in cords radiating from the central vein and separated by hepatic sinusoids (Figure 2). The low-dose group exhibited mild sinusoidal congestion, focal hepatocyte necrosis, and mild lymphoid infiltration in the portal area, with largely preserved architecture (Figure 3). In the high-dose group, hepatic architecture was markedly disrupted, with vascular congestion, extensive hepatocyte necrosis, and inflammatory cell infiltration (Figure 4).

Semi-quantitative histopathological scoring confirmed significant differences among the study groups. Sinusoidal/vascular congestion, hepatocyte necrosis, and inflammatory cell infiltration showed progressively higher severity scores from the control to the low- and high-dose groups. Architectural disruption was also significantly greater in ZnO NP-exposed animals than in controls. The semi-quantitative histopathological findings are summarized in Table 3.

### 2.5. Immunohistochemical Findings

#### 2.5.1. Bcl-2 Expression

**Figure 5:**
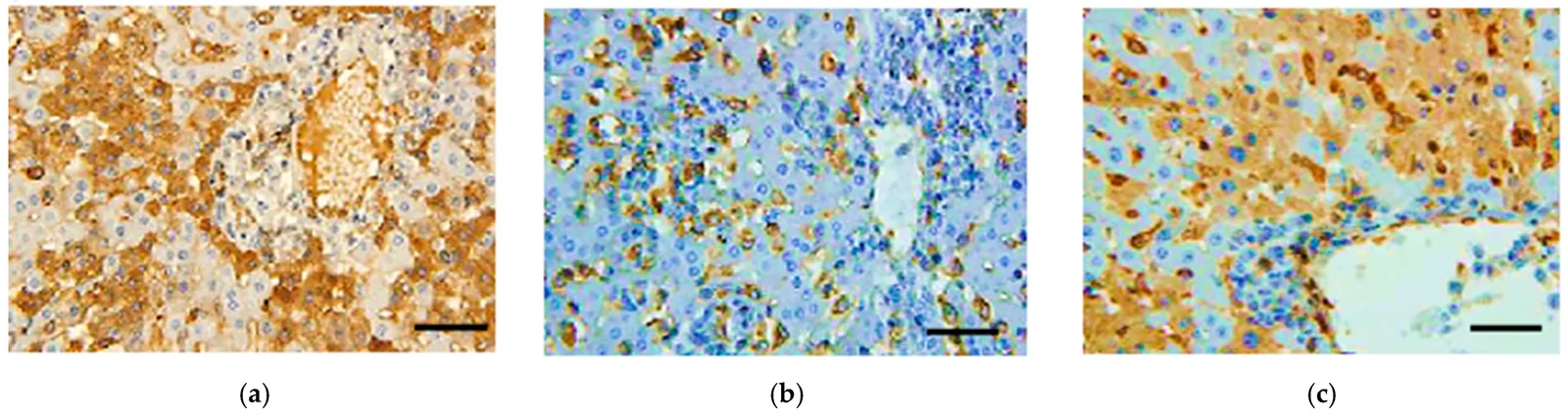
Bcl-2 immunohistochemical staining of rat liver following 28-day exposure to ZnO nanoparticles. (**a**) Control group; (**b**) low-dose group (30 mg/kg); (**c**) high-dose group (100 mg/kg). Bcl-2 immunoexpression decreased with increasing ZnO NP dose, with the lowest immunoreactivity observed in the high-dose group. IHC, ×400. Scale bar = 50 µm.

Immunohistochemical staining showed that Bcl-2 (an anti-apoptotic protein) immunoexpression decreased in a dose-dependent manner in ZnO NP-treated groups (Figure 5). The high-dose group showed markedly reduced Bcl-2 immunoreactivity compared with the low-dose and control groups, indicating a shift toward an apoptosis-permissive state.

#### 2.5.2. Caspase-3 Expression

**Figure 6:**
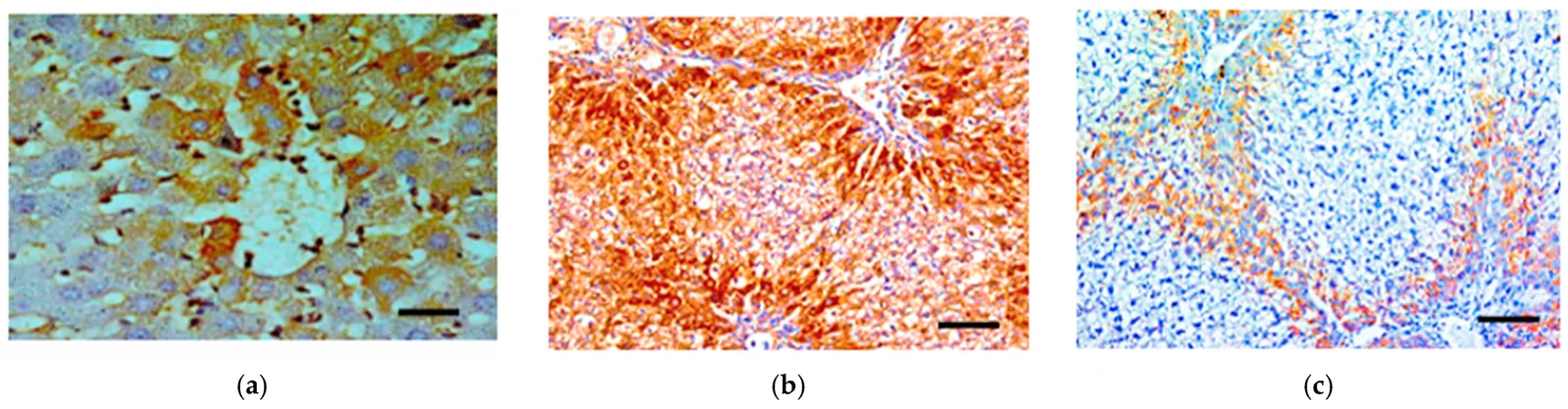
Caspase-3 immunohistochemical staining of rat liver following 28-day exposure to ZnO nanoparticles. (**a**) Control group; (**b**) low-dose group (30 mg/kg); (**c**) high-dose group (100 mg/kg). Caspase-3 immunoexpression increased with increasing ZnO NP dose, with the highest immunoreactivity observed in the high-dose group. IHC, ×400. Scale bar = 50 µm.

In contrast to Bcl-2, Caspase-3 (a pro-apoptotic executioner protein) immunoexpression increased in a dose-dependent manner in ZnO NP-treated groups (Figure 6). The high-dose group showed markedly increased Caspase-3 immunoreactivity compared with the low-dose and control groups, consistent with enhanced apoptotic activity.

#### 2.5.3. TNF-α Expression

**Figure 7:**
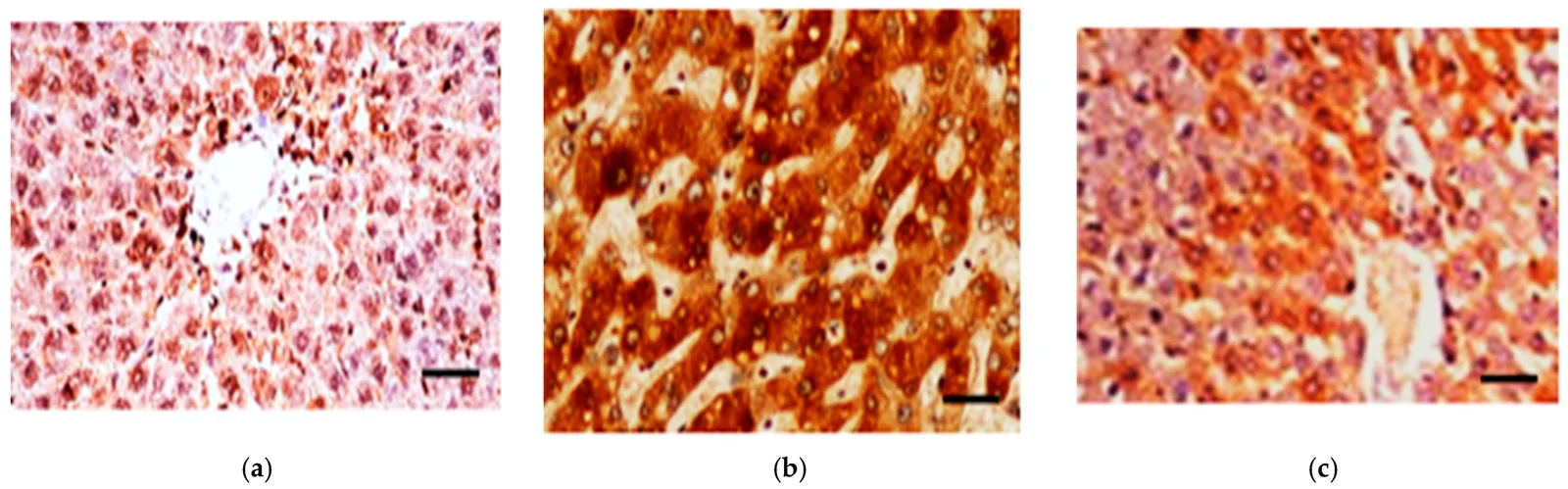
TNF-α immunohistochemical staining of rat liver following 28-day exposure to ZnO nanoparticles. (**a**) Control group; (**b**) low-dose group (30 mg/kg); (**c**) high-dose group (100 mg/kg). TNF-α immunoexpression increased with increasing ZnO NP dose, with the highest immunoreactivity observed in the high-dose group. IHC, ×400. Scale bar = 50 µm.

Similarly, TNF-α (a pro-inflammatory cytokine) immunoexpression increased in a dose-dependent manner in ZnO NP-treated groups (Figure 7). The high-dose group showed markedly increased TNF-α immunoreactivity compared with the low-dose and control groups, indicating an enhanced hepatic inflammatory response.

Semi-quantitative image analysis confirmed significant group differences in the immunoreactive area of Bcl-2, Caspase-3, and TNF-α. Bcl-2 immunoreactivity decreased progressively with ZnO NP exposure, whereas Caspase-3 and TNF-α immunoreactivity increased, with the greatest changes observed in the high-dose group. The quantitative IHC results are presented in Table 4.

### 2.6. Hepatic MicroRNA Expression (miR-122, miR-34a, miR-21)

**Figure 8:**
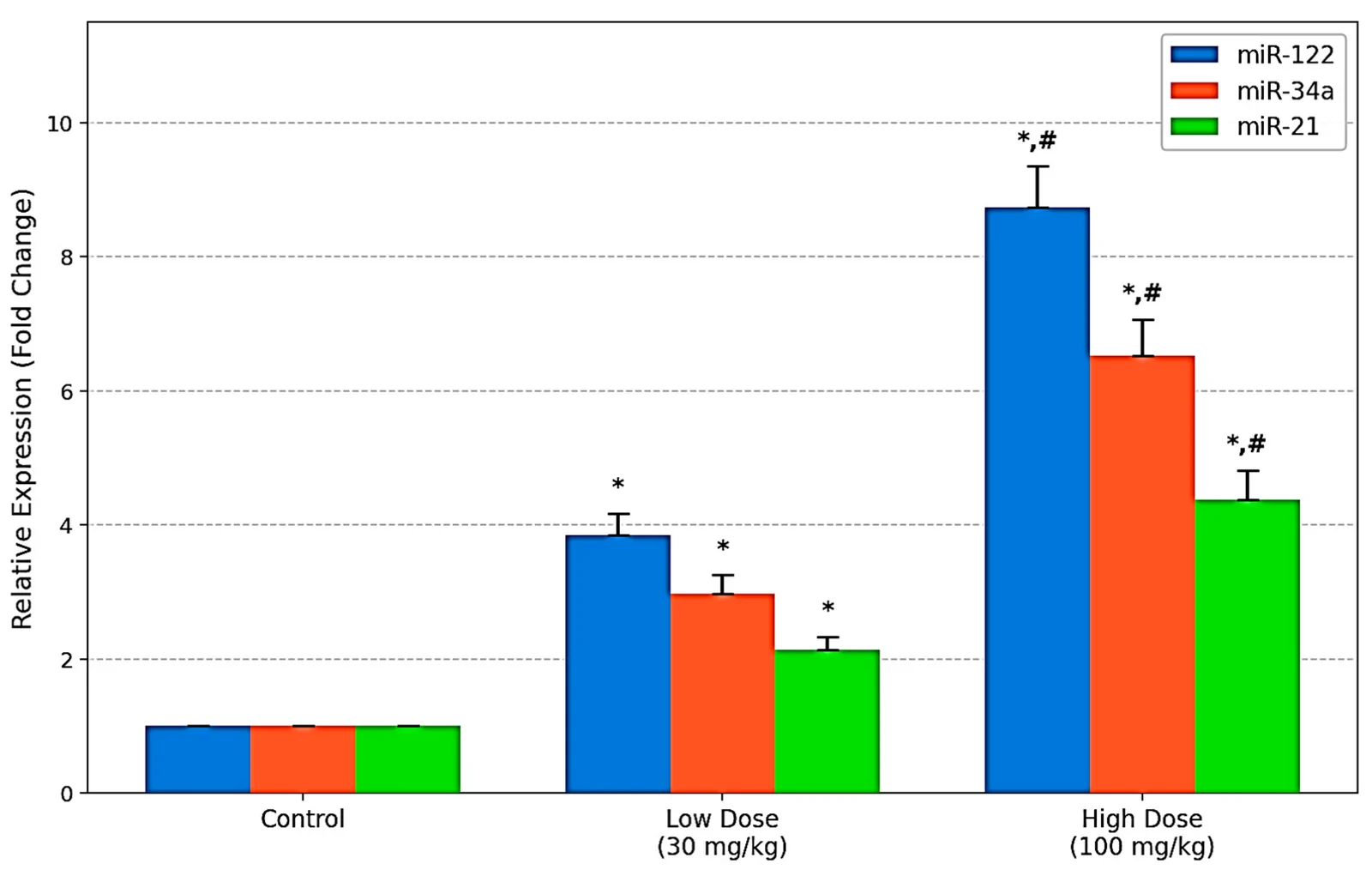
Hepatic microRNA expression (miR-122, miR-34a, miR-21) in rat liver following 28-day exposure to ZnO nanoparticles. All three microRNAs were significantly upregulated in a dose-dependent manner, with miR-122 showing the greatest increase. Values are expressed as mean fold change ± SEM (*n* = 20/group). * *p* < 0.05 vs. control; # *p* < 0.05 vs. low-dose group. One-way ANOVA followed by Tukey’s post hoc test.

Hepatic expression of miR-122, miR-34a, and miR-21 was significantly upregulated in a dose-dependent manner following ZnO NP exposure (Figure 8). miR-122, a liver-enriched microRNA and established biomarker of hepatocellular injury, showed the greatest increase, reaching 8.74-fold in the high-dose group. miR-34a, a pro-apoptotic and p53-responsive microRNA, increased to 6.53-fold, paralleling the elevated Caspase-3 and reduced Bcl-2 immunoexpression. miR-21, a stress- and inflammation-associated microRNA, increased to 4.38-fold, consistent with the elevated IL-6, HO-1, and TNF-α findings. These microRNA changes corroborated the biochemical, histopathological, and molecular results, collectively indicating progressive, dose-dependent hepatotoxicity.

### 2.7. Correlation Between Hepatic miRNA Expression and Markers of Liver Injury

**Figure 9:**
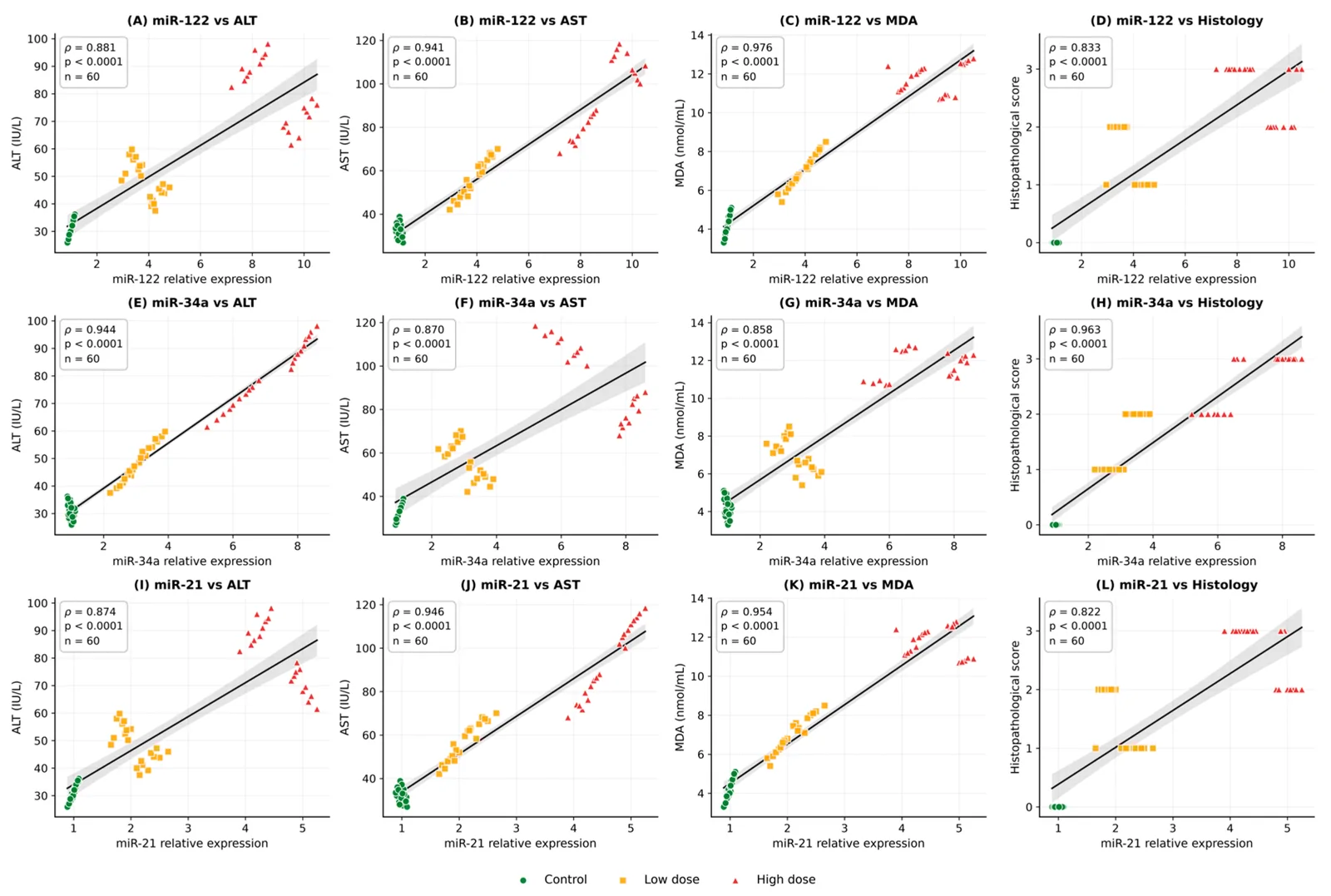
Relationships between hepatic miRNA expression and markers of liver injury in individual animals (*n* = 60). Scatter plots show the relationships of miR-122 (**A**–**D**), miR-34a (**E**–**H**), and miR-21 (**I**–**L**) relative expression with serum ALT, serum AST, hepatic MDA, and histopathological severity score. Each symbol represents one animal: green circles, control; orange squares, low-dose group (30 mg/kg); and red triangles, high-dose group (100 mg/kg). Solid lines represent least-squares trend lines and shaded areas indicate 95% confidence intervals for visualization. Spearman’s rank correlation coefficient (ρ), two-tailed *p*-value, and sample size are shown in each panel.

Spearman’s correlation analysis using individual-animal data (*n* = 60) demonstrated strong positive associations between hepatic miR-122, miR-34a, and miR-21 expression and markers of liver injury. miR-122 was positively correlated with ALT (ρ = 0.881), AST (ρ = 0.941), MDA (ρ = 0.976), and histopathological severity score (ρ = 0.833). miR-34a was likewise correlated with ALT (ρ = 0.944), AST (ρ = 0.870), MDA (ρ = 0.858), and histopathological severity score (ρ = 0.963). miR-21 showed positive correlations with ALT (ρ = 0.874), AST (ρ = 0.946), MDA (ρ = 0.954), and histopathological severity score (ρ = 0.822). All correlations were highly significant (all *p* < 0.0001) and remained significant after Benjamini–Hochberg correction for multiple testing. The complete correlation results are presented in Table 5, and the relationships with ALT, AST, MDA, and histopathological severity scores are illustrated in Figure 9.

## 3. Discussion

The present study demonstrates that oral administration of ZnO NPs to male rats for 28 days induces clear dose-dependent liver injury, with biochemical, histopathological, immunohistochemical, and molecular changes forming a coherent picture of progressive hepatotoxicity. The elevation of serum ALT and AST in both treated groups, greatest at 100 mg/kg, is consistent with the leakage of cytosolic and mitochondrial enzymes from hepatocytes with compromised membrane integrity, a pattern repeatedly documented for ZnO NPs at similar or lower doses [26,27,28] and, more broadly, in hepatotoxicant models in which mitochondrial dysfunction and oxidative stress accompany transaminase release [29]. A comparable dose–response for transaminase elevation was recently described in Sprague Dawley rats given ZnO NPs, in which hepatorenal enzyme activities rose dose-dependently in parallel with histological deterioration similar to that observed here [28]. Likewise, Al-Ragi et al. reported significant increases in AST and ALT, together with disruption of hepatic parenchymal architecture, following short-term ZnO NP exposure in albino mice [30].

The oxidative stress findings, namely the dose-dependent rise in MDA and the biphasic GSH response, align with the broader mechanistic scheme in which ZnO NPs exert their effects through reactive oxygen species generation. The increase in GSH observed at the low dose may reflect an adaptive antioxidant response to early oxidative challenge; however, this interpretation is not exclusive. ZnO dissolution and the resulting release of Zn^2+^ may also contribute to activation of Nrf2-dependent antioxidant pathways and enhanced glutathione synthesis. At the higher dose, the marked reduction in GSH is consistent with depletion or increased consumption of antioxidant reserves under a greater oxidative burden. A similar pattern of adaptive elevation followed by depletion has been reported in other models of ZnO NP hepatotoxicity, and in xenobiotic-induced liver injury more generally, in which low-to-moderate doses induced a defensive antioxidant response while higher doses depleted glutathione and increased lipid peroxidation in parallel with worsening hepatic function [7,28,31]. Combined administration of cerium and zinc oxide nanoparticles likewise increased hepatic MDA levels and reduced antioxidant capacity in a dose-related manner, consistent with the dose gradient used in the present design [32].

The biochemical picture was corroborated by histopathological examination. The high-dose group showed vascular congestion, frank necrosis, and loss of the typical cord-like arrangement of hepatocytes, whereas the low-dose group exhibited considerably milder congestion and focal necrotic changes. At higher ZnO NP burdens, architectural disintegration, including sinusoidal disruption, inflammatory cell infiltration, and hepatocellular necrosis, has been a consistent histological hallmark across multiple independent studies, providing external validation for the structural observations reported here [33,34,35]. Notably, the presence of portal lymphoid infiltration even at the low dose suggests that an early immune-mediated response precedes overt necrosis, a sequence consistent with reports describing immune cell recruitment as one of the earliest detectable hepatic responses to nanoparticle challenge, preceding more severe degenerative changes at higher doses [34].

The immunohistochemical findings add an apoptotic and inflammatory dimension to this injury process. The downregulation of Bcl-2, together with the marked upregulation of Caspase-3 and TNF-α immunoreactivity in the high-dose group, indicates a pro-apoptotic, pro-inflammatory hepatic milieu, consistent with the activation of both intrinsic and extrinsic apoptotic pathways. This pattern, in which the anti-apoptotic regulator Bcl-2 is suppressed while effector caspases and inflammatory cytokines are elevated, mirrors observations from nanoparticle and xenobiotic hepatotoxicity models, in which oxidative stress drives mitochondrial-mediated apoptosis through Bcl-2 suppression and concomitant activation of caspase cascades and nuclear factor kappa B (NF-κB)-driven cytokine production [34,35,36]. The increased TNF-α immunoexpression further supports the view that ZnO NP-induced hepatocyte injury is not purely oxidative but is potentiated by an inflammatory feedback loop, in which damaged hepatocytes and recruited immune cells perpetuate cytokine release, a mechanism increasingly implicated in ZnO NP and related metal-oxide nanoparticle hepatotoxicity, including through oxidative stress-driven pyroptotic signaling [32,37].

At the molecular level, the observed overexpression of IL-6 and HO-1 alongside SOD2 downregulation is mechanistically coherent. IL-6 induction reflects activation of inflammatory signaling cascades downstream of oxidative and cellular stress, while the pronounced increase in heme oxygenase-1, a stress-inducible enzyme largely governed by the nuclear factor erythroid 2-related factor 2 (Nrf2) antioxidant response pathway, likely represents a cytoprotective effort to mitigate the excess oxidative burden through heme catabolism and the generation of antioxidant by-products. The concomitant downregulation of SOD2, a key mitochondrial antioxidant enzyme, indicates that despite this HO-1 induction, the mitochondrial antioxidant defense system becomes progressively compromised with increasing ZnO NP dose, rendering hepatocytes more vulnerable to further oxidative insult. A similar combination of inflammatory cytokine induction, upregulation of stress-response genes, and suppression of antioxidant enzymes has been reported in rats exposed to ZnO NPs and related metal-oxide nanoparticles, with oxidative stress and inflammatory gene expression rising in parallel with a dose-dependent decline in endogenous antioxidant capacity [22,36].

The hepatic microRNA results extend these biochemical, histological, and molecular observations into the realm of post-transcriptional regulation. The dose-dependent upregulation of miR-122, a liver-enriched microRNA released from injured or necrotic hepatocytes, paralleled the rise in ALT and AST and is consistent with its established role as a sensitive tissue and circulating biomarker of hepatocellular injury that often precedes or tracks closely with conventional transaminase elevation in toxicological studies [23,25,38,39]. The upregulation of miR-34a, a well-characterized pro-apoptotic transcriptional target of p53, aligns with the increased Caspase-3 immunoreactivity and decreased Bcl-2 expression observed histologically, consistent with an association between p53-related stress signaling and apoptotic activation in ZnO-exposed liver tissue [24].

The upregulation of miR-21, previously described in hepatic stress settings as a component of a compensatory pro-survival and pro-inflammatory response, is consistent with the increased HO-1, IL-6, and TNF-α expression observed here, and suggests that this microRNA may participate in modulating the balance between cell survival and inflammatory signaling during sustained nanoparticle-induced hepatic stress [24]. Collectively, the concordant dysregulation of these three microRNAs and their directional agreement with the biochemical and molecular markers of injury are in line with the broader literature linking miR-122, miR-34a, and miR-21 to hepatocellular injury, apoptosis, and inflammatory liver disease in both toxicological and clinical settings [23,24].

Taken together, the biochemical, histopathological, immunohistochemical, molecular, and microRNA findings are consistent with a dose-dependent hepatic response following ZnO NP exposure. Oxidative stress appears to be an important component of this response and is accompanied by inflammatory signaling, impaired mitochondrial antioxidant defense, apoptotic activation, and altered hepatic microRNA expression. However, because a soluble zinc-ion comparator was not included, the relative contribution of particulate ZnO and released Zn^2+^ ions cannot be distinguished in the present study.

The correlation analysis further strengthened the relationship between hepatic microRNA dysregulation and the severity of liver injury. All three investigated microRNAs showed strong positive correlations with serum ALT and AST, hepatic MDA, and histopathological severity scores. The strongest associations included miR-122 with MDA (ρ = 0.976), miR-34a with histopathological severity (ρ = 0.963), and miR-21 with MDA (ρ = 0.954). These findings indicate that increases in hepatic miR-122, miR-34a, and miR-21 occurred in parallel with biochemical, oxidative, and structural evidence of increasing hepatic injury. Nevertheless, these correlations demonstrate association rather than direct mechanistic causation.

### Study Limitations

Several limitations should be acknowledged. The study evaluated only two doses and a single 28-day exposure period; therefore, intermediate doses and longer exposure durations were not assessed. The reversibility of the observed injury after cessation of exposure was not examined. Finally, validated human (HSA) microRNA assays were used based on the full sequence conservation of these microRNAs between human and rat; although widely accepted, species-specific validation would further reinforce the findings. The present study was observational at the molecular level and did not include functional manipulation of the investigated microRNAs. Therefore, direct regulatory effects of miR-122, miR-34a, or miR-21 on downstream target genes were not established. Future mechanistic studies using miRNA inhibition or overexpression in hepatocyte models are warranted. Gene-expression normalization was performed using GAPDH as the single endogenous reference gene. Because an additional reference gene such as β-actin or 18S rRNA was not evaluated, potential treatment-related variation in GAPDH expression cannot be completely excluded. Future studies should validate reference-gene stability using multiple candidate housekeeping genes under the experimental conditions. Finally, the particle size and purity were based on the manufacturer’s specifications, and no independent physicochemical characterization of the administered ZnO preparation was performed. Consequently, the hydrodynamic particle size, polydispersity, zeta potential, aggregation state in the dosing vehicle, morphology, and crystalline phase could not be independently confirmed. This limits the ability to relate the observed biological responses to precisely defined physicochemical characteristics of the administered material. In addition, the experimental design did not include a soluble zinc-salt comparator group. Therefore, the present study cannot distinguish the relative contribution of the particulate ZnO form from that of dissolved Zn^2+^ ions to the observed hepatic effects. Future studies should include an equivalent-dose soluble zinc control to address this distinction.

## 4. Materials and Methods

### 4.1. Ethical Approval

All animal procedures were conducted in accordance with the ethical principles and national guidelines for the care and use of laboratory animals in scientific research, and were approved by the Scientific Research Ethics Committee of the College of Science, University of Al-Qadisiyah (project no. SCI-2025-063; approval no. REC/201/2025, dated 16 December 2025).

All efforts were made to minimize animal suffering and to reduce the number of animals used, in accordance with the 3Rs principles. Throughout the exposure period, the animals were monitored daily by a qualified person for body weight, food and water intake, and any clinical signs of pain or distress. At the end of the experiment, all animals were deeply anesthetized before sacrifice, and euthanasia was performed rapidly by trained personnel under full anesthesia to ensure a humane death.

### 4.2. Animals and Experimental Design

Sixty healthy male Wistar albino rats, seven weeks old and weighing 180–210 g at the beginning of the experiment, were obtained from the Animal House of the College of Science, University of Al-Qadisiyah. The animals were housed in standard polypropylene cages under controlled conditions of 22 ± 2 °C, 50–60% relative humidity, and a 12 h light/12 h dark cycle, with free access to standard laboratory chow and water. The animals were allowed to acclimatize for seven days before the initiation of treatment, after which they were randomly allocated into three equal groups (*n* = 20 each):

- Control group: received the vehicle (distilled water) alone at 10 mL/kg body weight daily for 28 days.
- Low-dose group: received 30 mg/kg body weight of ZnO NPs daily for 28 days (3 mg/mL suspension, 10 mL/kg).
- High-dose group: received 100 mg/kg body weight of ZnO NPs daily for 28 days (10 mg/mL suspension, 10 mL/kg).

All treatments were administered once daily by oral gavage for 28 consecutive days.

### 4.3. Preparation and Administration of ZnO NPs

Zinc oxide nanoparticles (ZnO NPs; stock no. 8431UV) were purchased from SkySpring Nanomaterials, Inc. (Houston, TX, USA). According to the manufacturer’s specifications, the ZnO material had a nominal primary particle size of 50 nm and a purity of 99.9%. Independent physicochemical characterization, including dynamic light scattering (DLS), zeta-potential analysis, transmission electron microscopy (TEM), and X-ray diffraction (XRD), was not performed as part of the original experimental design. Therefore, the hydrodynamic size, polydispersity, aggregation state, surface charge, morphology, and crystalline phase of the administered ZnO suspension were not independently verified. Before administration, the ZnO NPs were suspended in distilled water and dispersed by ultrasonication for 10 min. The suspension was freshly prepared for each dosing session and administered by oral gavage immediately after sonication to minimize settling prior to administration.

### 4.4. Sample Collection

At the end of the 28-day exposure period, animals were anesthetized with ether and sacrificed by cervical dislocation. Blood samples were collected by cardiac puncture, and sera were separated for biochemical analysis. Livers were rapidly excised, washed in cold phosphate-buffered saline (PBS), and processed for histopathological, immunohistochemical, and molecular analyses. Tissue portions designated for RNA extraction were snap-frozen and stored at −80 °C until use.

### 4.5. Biochemical Analyses

Serum alanine aminotransferase (ALT) and aspartate aminotransferase (AST) were measured using commercial reagents (ALT/GPT plus, cat. no. 11832; AST/GOT plus, cat. no. 11830; IFCC method, manual format; BioSystems S.A., Barcelona, Spain). Hepatic oxidative stress markers, malondialdehyde (MDA) and reduced glutathione (GSH), were assessed using commercial micro-method kits (MDA Content Assay Kit, cat. no. BC0025; Reduced Glutathione Content Assay Kit, cat. no. BC1175; Solarbio Science & Technology Co., Beijing, China), according to the manufacturers’ instructions.

### 4.6. Histopathological Examination

Liver specimens were fixed in 10% neutral buffered formalin, processed, embedded in paraffin, sectioned, and stained with hematoxylin and eosin (H&E) for histological evaluation of the three groups [20]. Sections were examined and photographed using an Olympus BX53 light microscope fitted with a DP74 digital camera (Olympus, Tokyo, Japan).

Histopathological alterations were additionally evaluated using a semi-quantitative severity scoring system. Sinusoidal/vascular congestion, hepatocyte necrosis, inflammatory cell infiltration, and architectural disruption were scored on a 0–3 ordinal scale, where 0 indicated absence of the lesion, 1 mild involvement, 2 moderate involvement, and 3 severe involvement. Scores were assigned for all animals in each group and were subsequently used for statistical comparisons. Histopathological scoring was performed blinded to treatment-group allocation.

### 4.7. Immunohistochemistry

Paraffin-embedded liver sections were deparaffinized and rehydrated through graded ethanol. Heat-induced antigen retrieval was performed in 10 mM citrate buffer (pH 6.0) in a microwave oven at 95–98 °C for 15 min; the sections were then left to cool in the retrieval buffer for 20 min at room temperature and washed three times in PBS (2 min each). Endogenous peroxidase activity was quenched with 3% hydrogen peroxide for 10 min, followed by three PBS washes, and non-specific binding was blocked with ready-to-use normal goat blocking buffer for 30 min at 37 °C, the excess being drained rather than washed off.

Sections were incubated overnight at 4 °C with rabbit polyclonal primary antibodies against B-cell lymphoma 2 (Bcl-2; cat. no. E-AB-60012, 1:100), Caspase-3 (cat. no. E-AB-63510, 1:100) or tumor necrosis factor alpha (TNF-α; cat. no. E-AB-33121, 1:200) (Elabscience Biotechnology Inc., Houston, TX, USA). The following day they were equilibrated at 37 °C for 30 min and washed three times in PBS. Immunodetection was carried out with the 2-Step Plus Poly-HRP Anti-Rabbit IgG Detection System with DAB (cat. no. E-IR-R215, Elabscience): ready-to-use polyperoxidase-conjugated anti-rabbit IgG was applied for 20 min at room temperature, and immunoreactivity was visualized with freshly prepared 3,3′-diaminobenzidine (DAB) working solution for 3–5 min under microscopic control, the reaction being terminated by rinsing in deionized water. Sections were counterstained with Mayer’s hematoxylin for 1 min, blued in running tap water, dehydrated through graded ethanol, cleared in xylene and mounted in permanent mounting medium. Negative controls were processed identically in every staining run, with the primary antibody omitted and replaced by antibody diluent. For semi-quantitative immunohistochemical analysis, digital images were analyzed using ImageJ software (version 1.54g; National Institutes of Health, Bethesda, MD, USA). For each animal, five non-overlapping high-power fields were evaluated under standardized imaging conditions. The percentage of DAB-positive immunoreactive area was quantified for Bcl-2, Caspase-3, and TNF-α, and the mean value of the analyzed fields was used as the animal-level observation for statistical analysis.

### 4.8. RNA Extraction

Total RNA, including the small RNA fraction, was extracted from frozen liver tissue (approximately 50 mg per rat) using the miRNeasy Mini Kit (Qiagen, Hilden, Germany), a phenol/guanidine-based extraction combined with silica-membrane purification, according to the manufacturer’s protocol [40]. RNA concentration and purity were assessed spectrophotometrically using a NanoDrop One instrument (Thermo Fisher Scientific, Waltham, MA, USA); only samples with an A260/A280 ratio of 1.8–2.0 were used in downstream analyses.

### 4.9. Gene Expression Analysis (RT-qPCR)

Complementary DNA (cDNA) was synthesized from total RNA using a High-Capacity cDNA Reverse Transcription Kit (Bioneer Corp., Daejeon, Republic of Korea) according to the manufacturer’s instructions. The relative expression of IL-6, HO-1, and SOD2 was quantified by RT-qPCR using PowerUp SYBR Green Master Mix (Applied Biosystems, Foster City, CA, USA) with gene-specific primers. GAPDH served as the endogenous reference gene for normalization. Relative expression was calculated using the 2^−ΔΔCt^ method [41], with the control group as the calibrator, and reactions were performed in triplicate. The primers for IL-6, SOD2, HO-1, and GAPDH were designed based on the corresponding *Rattus norvegicus* reference sequences retrieved from the NCBI database, and their specificity was verified using BLAST (BLASTN version 2.15.0; National Center for Biotechnology Information, Bethesda, MD, USA). The primers were synthesized by Macrogen Inc. (Seoul, Republic of Korea). The primer sequences and accession numbers are listed in Table 6.

### 4.10. MicroRNA Quantification (RT-qPCR)

For microRNA analysis, cDNA was synthesized from total RNA using the TaqMan Advanced miRNA cDNA Synthesis Kit (Applied Biosystems, Foster City, CA, USA). The expression levels of miR-122-5p, miR-34a-5p, and miR-21-5p (hereafter miR-122, miR-34a, and miR-21) were quantified using TaqMan Advanced miRNA Assays (Applied Biosystems), as listed in Table 7, on a QuantStudio 5 Real-Time PCR System (Applied Biosystems, Foster City, CA, USA) under the following conditions: initial denaturation at 95 °C for 10 min, followed by 40 cycles of 95 °C for 15 s and 60 °C for 60 s. Because the mature sequences of miR-122-5p, miR-34a-5p, and miR-21-5p are fully conserved between human and rat, validated human () TaqMan Advanced miRNA Assays were used for quantification in rat liver tissue. The small nuclear RNA U6 (U6 snRNA) served as the endogenous reference control. Relative expression was calculated using the 2^−ΔΔCt^ method [41], with the control group set as the calibrator (1.00). All experiments followed the MIQE guidelines for qPCR reporting [42]. Reactions were performed in triplicate.

### 4.11. Statistical Analysis

Results were expressed as mean ± standard error of the mean (SEM) (*n* = 20 per group). Normality of distribution was verified using the Shapiro–Wilk test and homogeneity of variance using Levene’s test. Differences among groups were analyzed by one-way analysis of variance (ANOVA) followed by Tukey’s post hoc test. Quantitative immunohistochemical data were analyzed by one-way ANOVA followed by Tukey’s post hoc multiple-comparison test. Histopathological severity scores were treated as ordinal data and are presented as median (interquartile range). Group differences were assessed using the Kruskal–Wallis test followed by Dunn’s post hoc multiple-comparison test. Correlations between hepatic miR-122, miR-34a, and miR-21 expression and markers of liver injury (ALT, AST, MDA, and histopathological severity scores) were assessed using individual-animal data (*n* = 60). Normality of the continuous variables was evaluated using the Shapiro–Wilk test. Because all six continuous variables deviated significantly from a normal distribution in the pooled dataset (all *p* < 0.001), Spearman’s rank correlation coefficient (ρ) was used throughout. Spearman’s correlation was also used for histopathological severity scores because these were ordinal data. Two-tailed *p*-values and 95% confidence intervals for ρ were calculated. Because 12 correlations were tested, *p*-values were additionally adjusted using the Benjamini–Hochberg false-discovery-rate procedure. A value of *p* < 0.05 was considered statistically significant. Primary statistical analyses were performed using SPSS software, version 27 (IBM SPSS Statistics, Armonk, NY, USA), whereas the correlation analyses and multiple-testing correction were performed using Python 3.12.

## 5. Conclusions

This study demonstrates that subacute oral exposure to the administered ZnO nanoparticle preparation was associated with dose-dependent hepatotoxicity in rats, accompanied by oxidative stress, inflammation, mitochondrial antioxidant dysfunction, and apoptosis. Importantly, hepatic miR-122, miR-34a, and miR-21 were concurrently upregulated and showed strong positive correlations with biochemical, oxidative, and histopathological indicators of liver injury, supporting their potential value as candidate biomarkers of ZnO NP-associated hepatotoxicity.

These findings suggest that integrating conventional toxicological markers with microRNA profiling offers a more comprehensive assessment of nanoparticle-induced liver injury. Further studies are warranted to investigate the long-term effects, reversibility, and regulatory roles of these microRNAs, and to evaluate their potential as early diagnostic and prognostic biomarkers of nanoparticle-related hepatic toxicity.

## Figures and Tables

**Table 1:** Serum liver enzyme levels in rats following 28-day exposure to ZnO nanoparticles.

Parameter	Control Group	Low-Dose Group	High-Dose Group
ALT (IU/L)	31.26 ± 2.5	48.27 ± 5.1 *	75.48 ± 12.3 *^,#^
AST (IU/L)	32.47 ± 4.1	59.38 ± 8.6 *	85.57 ± 18.9 *^,#^

Values are expressed as mean ± SEM (*n* = 20/group). * *p* < 0.05 vs. control; ^#^ *p* < 0.05 vs. low-dose group. One-way ANOVA followed by Tukey’s post hoc test.

**Table 2:** Hepatic oxidative stress markers in rats following 28-day exposure to ZnO nanoparticles.

Parameter	Control Group	Low-Dose Group	High-Dose Group
MDA (nmol/mL)	4.21 ± 0.55	6.83 ± 0.91 *	11.52 ± 0.31 *^,#^
GSH (μmol/mL)	3.52 ± 0.9	6.68 ± 0.7 *	2.03 ± 0.5 *^,#^

Values are expressed as mean ± SEM (*n* = 20/group). * *p* < 0.05 vs. control; ^#^ *p* < 0.05 vs. low-dose group. One-way ANOVA followed by Tukey’s post hoc test.

**Table 3:** Semi-quantitative histopathological scores of rat liver following 28-day exposure to zinc oxide nanoparticles.

Histopathological Parameter	Control	Low-Dose (30 mg/kg)	High-Dose (100 mg/kg)	*p*-Value
Sinusoidal/vascular congestion	0.0 (0.0–0.0) ^a^	1.0 (1.0–1.5) ^b^	3.0 (2.5–3.0) ^c^	0.0117
Hepatocyte necrosis	0.0 (0.0–0.0) ^a^	1.5 (1.0–2.0) ^b^	3.0 (2.0–3.0) ^c^	0.0352
Inflammatory cell infiltration	0.0 (0.0–0.0) ^a^	2.0 (2.0–2.5) ^b^	3.0 (2.5–3.0) ^c^	0.0225
Architectural disruption	0.0 (0.0–0.0) ^a^	1.5 (1.0–2.0) ^b^	2.0 (1.5–2.5) ^b^	0.0119

Values are expressed as median (interquartile range, IQR) of semi-quantitative histopathological scores (0–3) for each group (*n* = 20 rats/group). Data were analyzed using the Kruskal–Wallis test followed by Dunn’s post hoc multiple-comparison test. Groups not sharing the same superscript letter (a, b, c) differ significantly at *p* < 0.05.

**Table 4:** Quantitative immunohistochemical analysis of Bcl-2, Caspase-3, and TNF-α expression in rat liver following 28-day exposure to zinc oxide nanoparticles.

Marker	Control	Low-Dose (30 mg/kg)	High-Dose (100 mg/kg)	*p*-Value
Bcl-2 (% positive area)	4.885 ± 0.321 ^c^	3.962 ± 0.523 ^b^	2.051 ± 0.052 ^a^	0.0145
Caspase-3 (% positive area)	1.251 ± 0.0221 ^a^	3.014 ± 0.0395 ^b^	5.001 ± 0.014 ^c^	0.0325
TNF-α (% positive area)	3.014 ± 0.095 ^a^	5.014 ± 0.054 ^b^	6.595 ± 0.065 ^c^	0.0114

Values are expressed as mean ± SD of the percentage of immunopositive area per group (*n* = 20 rats/group). Data were analyzed using one-way ANOVA followed by Tukey’s post hoc multiple-comparison test. Groups not sharing the same superscript letter (a, b, c) differ significantly at *p* < 0.05.

**Table 5:** Correlations between hepatic miRNA expression and markers of liver injury using individual-animal data (*n* = 60).

Correlation Pair	Test Used	Correlation Coefficient	95% CI	*p*-Value	N
miR-122 vs. ALT	Spearman	ρ = 0.881	0.791 to 0.933	<0.0001	60
miR-122 vs. AST	Spearman	ρ = 0.941	0.892 to 0.968	<0.0001	60
miR-122 vs. MDA	Spearman	ρ = 0.976	0.955 to 0.987	<0.0001	60
miR-122 vs. histopathological score	Spearman	ρ = 0.833	0.715 to 0.905	<0.0001	60
miR-34a vs. ALT	Spearman	ρ = 0.944	0.898 to 0.970	<0.0001	60
miR-34a vs. AST	Spearman	ρ = 0.870	0.774 to 0.927	<0.0001	60
miR-34a vs. MDA	Spearman	ρ = 0.858	0.755 to 0.920	<0.0001	60
miR-34a vs. histopathological score	Spearman	ρ = 0.963	0.932 to 0.980	<0.0001	60
miR-21 vs. ALT	Spearman	ρ = 0.874	0.779 to 0.929	<0.0001	60
miR-21 vs. AST	Spearman	ρ = 0.946	0.901 to 0.971	<0.0001	60
miR-21 vs. MDA	Spearman	ρ = 0.954	0.915 to 0.975	<0.0001	60
miR-21 vs. histopathological score	Spearman	ρ = 0.822	0.698 to 0.898	<0.0001	60

ρ, Spearman’s rank correlation coefficient; CI, confidence interval. All correlations were calculated using individual-animal data (*n* = 60). All 12 correlations remained statistically significant after Benjamini–Hochberg false-discovery-rate correction.

**Table 6:** Primer sequences used for gene expression analysis by RT-qPCR (SYBR Green).

Gene	Primer Sequence (5′ → 3′)	Amplicon Size	Accession No.
IL-6	F: CTTCCAGCCAGTTGCCTTCT R: TGGTCTGTTGTGGGTGGTAT	79 bp	NM_012589.2
SOD2	F: CAGACCTGCCTTACGACTATGG R: CTCGGTGGCGTTGAGATTGTT	150 bp	NM_017051.2
HO-1	F: CTGACAGAAGGAGGCCAAGA R: CTGACAGATGCCGAGTGTTC	130 bp	NM_012580.2
GAPDH	F: GACATGCCGCCTGGAGAAAC R: AGCCCAGGATGCCCTTTAGT	202 bp	AB017801

F, forward primer; R, reverse primer.

**Table 7:** TaqMan Advanced miRNA assays used for microRNA quantification by RT-qPCR.

miRNA Target	Assay Type	TaqMan Assay ID	Reference Gene
miR-122-5p	TaqMan Advanced miRNA Assay (hsa)	478887_mir	U6 snRNA
miR-34a-5p	TaqMan Advanced miRNA Assay (hsa)	478886_mir	U6 snRNA
miR-21-5p	TaqMan Advanced miRNA Assay (hsa)	477975_mir	U6 snRNA

## Data Availability

The original contributions presented in this study are included in the article. Further inquiries can be directed to the corresponding author.

## Abbreviations

The following abbreviations are used in this manuscript:

ZnO NPs	Zinc oxide nanoparticles
ALT	Alanine aminotransferase
AST	Aspartate aminotransferase
MDA	Malondialdehyde
GSH	Reduced glutathione
SOD2	Superoxide dismutase 2 (mitochondrial)
Cu/Zn-SOD	Copper–zinc superoxide dismutase
HO-1	Heme oxygenase-1
IL-6	Interleukin-6
TNF-α	Tumor necrosis factor alpha
Bcl-2	B-cell lymphoma 2
Nrf2	Nuclear factor erythroid 2-related factor 2
NF-κB	Nuclear factor kappa B
miRNA	MicroRNA
RT-qPCR	Reverse transcription–quantitative polymerase chain reaction
GAPDH	Glyceraldehyde-3-phosphate dehydrogenase
U6 snRNA	U6 small nuclear RNA
H&E	Hematoxylin and eosin
IHC	Immunohistochemistry
DAB	3,3′-Diaminobenzidine
PBS	Phosphate-buffered saline
cDNA	Complementary DNA
SEM	Standard error of the mean
ANOVA	Analysis of variance
MIQE	Minimum Information for Publication of Quantitative Real-Time PCR Experiments
